# Comparison of Antifungal Efficacy of Zataria Multiflora and Nystatin for Treatment of Denture Stomatitis: A Randomized Clinical Trial

**DOI:** 10.30476/DENTJODS.2020.84181.1069

**Published:** 2021-03

**Authors:** Elnaz Gonoudi, Masoud Rezai, Taraneh Farrokhnia, Mehdi Goudarzi, Alireza Sima

**Affiliations:** 1 Dept. of Oral Medicine, Faculty of Dentistry, Tehran Medical Sciences, Islamic Azad University, Tehran, Iran; 2 Dept. of Microbiology, School of Medicine, Shahid Beheshti University of Medical Sciences, Tehran, Iran; 3 Digestive Disease Research Institute, Tehran University of Medical Sciences, Tehran, Iran

**Keywords:** *Candida albicans*, Denture stomatitis, Nystatin, Zataria multiflora

## Abstract

**Statement of the Problem:**

Zataria multiflora (ZM) is a thyme-like plant that belongs to the Lamiaceae family. It is native to the center and south of Iran, Pakistan, and Afghanistan. Evidence shows that ZM contains thymol and carvacrol and is therefore, effective for the treatment of many conditions especially fungal infections. Oral candidiasis is the most common opportunistic infection of the oral mucosa that plays a role in the development of denture stomatitis.

**Purpose:**

This study aimed to compare the antifungal efficacy of ZM and nystatin suspension for the treatment of denture stomatitis.

**Materials and Method:**

This single-blind clinical trial evaluated 28 patients (> 18 years old) suffering from type II or III denture stomatitis. Patients were divided into two groups. The control group used nystatin suspension while the case group used ZM drop. The number of *Candida albicans* (*C. albicans*) colony-forming units (CFUs) and erythema of the palate were evaluated at baseline and at 14 days after treatment. Data were analyzed using SPSS version 11 via Student’s t test and repeated measure ANOVA.

**Results:**

The results showed similar efficacy of nystatin and ZM in the reduction of *C.albicans* CFUs compared to the baseline value (*p*= 0.593). Both medications significantly decreased the colony count (*p*< 0.001). Nystatin and ZM had similar efficacy for the reduction of erythema as well (*p*= 0.256) and both caused a significant reduction in erythema of the palate (*p*<0.001).

**Conclusion:**

ZM drop was as effective as the nystatin drop in the resolution of erythema of the palate and reduction of *C. albicans* colony count.

## Introduction

Oral candidiasis is the most common opportunistic infection affecting the oral mucosa. In most cases, the infection is caused by the *Candida albicans* (*C. albicans*) yeast. It has a prevalence of 35% as a member of the normal flora. Under certain circumstances, *C. albicans* may become invasive. A significant association has been noted between oral candidiasis and presence of local predisposing factors (such as wearing a denture, smoking, and use of inhaled or topical steroids) and some systemic conditions (immune system and endocrine status), causing transformation of *C. albicans* to its pathogenic variant [ 1 ]. Biofilms containing *C. albicans* play a role in the development of denture stomatitis. Denture stomatitis is the most common and the most important clinical condition occurring in denture wearers [ 2 ]. It has a multifactorial etiology. Long-term use of denture is the most important risk factor for the colonization of candida species on the mucosal surface of denture and development of oral candidiasis, which is affected by exogenous and endogenous factors [ 3 ]. At present, antifungal medications play a primary role in the treatment of denture stomatitis. However, they have side effects and are associated with a high risk of recurrence. Antifungal agents mainly belong to the family of azoles or polyenes. Polyenes such as nystatin are the primary choice for the treatment of primary oral candidiasis [ 2 ]. However, they have some side effects such as bitter taste, poor acceptance by patients, mucosal irritation, and nausea [ 4 ]. 

There is a growing trend towards the use of herbal medications worldwide. Affordability, availability, fewer side effects, not causing microbial resistance, and significant therapeutic effects are among the most important properties of herbal medications [ 5 - 7 ]. Zataria multiflora (ZM) is a thyme-like medicinal herb, and the contemporary pharmacological studies have confirmed its anti-inflammatory, analgesic, antispasmodic, and antimicrobial properties. ZM contains thymol and carvacrol and it is used for the treatment of many conditions particularly fungal, bacterial, and parasitic infections. Some studies have reported the antifungal effects of ZM oil against candida [ 5 , 8 ]. In 2018, de Oliveira Santos *et al*. [ 9 ] showed in a brief review that ZM has the best antifungal effect among various herbal essential oils. Nazzaro *et al*. [ 10 ] also noted similar results in another study. Considering the scarcity of investigations on the antifungal efficacy of ZM and its comparison with the commonly prescribed synthetic antifungal agents, this study aimed to assess and compare the antifungal effects of ZM and nystatin. 

## Materials and Method

**Figure 1:**
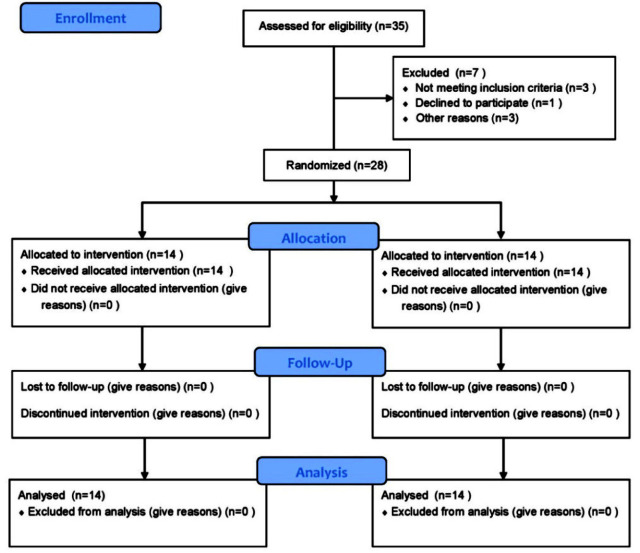
CONSORT Flow Diagram

This study is a randomized clinical trial and single-blinded design. After obtaining ethical approval (IR.IAU, DENTAL.REC.1395, 35) and registration in the Iranian Registry of Clinical Trials (IRCT) (IRCT201706183461 9 N1) (2017-09-24), written informed consent was obtained from all patients before their enrollment. Patients were selected among those presenting to the dental clinic of the Islamic Azad University of Tehran. A total of 28 patients over 18 years of age suffering from type II or III denture stomatitis were selected (Figure 1). Denture stomatitis has three types. Type II presents an extensive erythematous mucosa under the denture. Type III is characterized by the presence of a granular mucosa at the center of the palate in addition to the involved areas mentioned for type II [ 11 ]. The exclusion criteria were patients with hepatic cirrhosis, renal insufficiency, thyroid dysfunction (contraindication for use of ZM), diabetes mellitus, xerostomia, hypoparathyroidism, pregnancy or nursing, immunocompromised patients, chemotherapy, radiotherapy, and patients who had taken antibiotics or antifungal agents in the past 4 weeks [ 12 - 13 ]. Patients were questioned about the duration of using removable denture, its cleaning protocol (water, toothbrush, denture cleansing agents), frequency of cleansing the denture (zero, once a week, twice a week, three times a week, more than three times a week) and that whether they remove it at night. The presence of erythema of the palate was also recorded. For this purpose, the affected area was outlined with a copying pencil and its surface area was measured using a 5mm squared transparent grid and reported in square millimeters. Eventually, the patients were requested to rinse their mouth with drinking water. Then a microbial sample was taken from the erythematous area from hard palate using a sterile swab to count the *C. albicans* colony forming units (CFUs) before treatment [ 12 ]. The patients were then divided into two groups of 14, using stratified block randomization since patients had type II or III denture stomatitis and we wanted to ensure an equal number of both types in the two groups. 

In the control group, patients were requested to rinse 40 drops of 100,000-unit nystatin suspension (Emad Pharmaceuticals, Tehran, Iran) for 2 minutes four times a day for two weeks [ 13 ]. In the case group, patients were requested to rinse one teaspoon (5mL) of ZM essential oil 0.05 % (Gastroli; Bridge Essence) containing 5mg/mL thymol and carvacrol for 2minutes four times a day for two weeks [ 12 ]. It should be noted that the medications were given to patients by another person not involved in the study and according to computer-generated random numbers (2 series from 1 to 28 for types II and III denture stomatitis).

Patients were instructed to remove the denture, rinse their mouth with water, and then use the medication. In addition, they were instructed to use their denture only for eating. Cleaning the denture after meals with a soft toothbrush without toothpaste, removal of the denture at night, and its immersion in water overnight were also emphasized [ 14 ]. Since nystatin interferes with chlorhexidine, patients were requested not to immerse their dentures in chlorhexidine during the study period [ 15 ]. 

Patients were examined at the end of day 14. A microbial sample was taken from the palate using a sterile swab and the presence/absence of erythema was recorded. The *C. albicans* colony count was recorded again. The *C. albicans* colonies were counted using the method as follows. Microbial samples taken from the palate were placed in sterile saline and transferred to the microbiology lab within 2 hours. The test tube containing the swab and saline was shaken on a shaker and then 0.1 mL of the homogenous solution was taken by a sampler, cultured on Sabouraud dexterous agar plate, and incubated for 24 hours. Next, the presence of *C. albicans* colonies was confirmed using the germ tube test and they were counted under a light microscope (SE, Nikon, Japan). In the germ tube test, one colony was chosen and placed in 2 cc of human serum. After 2 hours, a slide was prepared of the serum, and the presence of *C. albicans* colonies was microscopically confirmed [ 16 ].

### Statistics analysis

We used Student’s t test for comparison of quantitative data with normal distribution between two groups and repeated measure ANOVA for comparison more than two groups or to compare a group in more than one position or time, respectively. Data were analyzed with SPSS 11 (IBM Corp.) and Statistical significance was accepted as *p*< 0.05.

## Results

A total of 7 females (25%) and 21 males (75%) participated in this study. The nystatin group included 5 females and 9 males with a mean age of 60.93±13.04 years (range 38 to 82 years) while the ZM group included 2 females and 12 males with a mean age of 55.86± 10.04 years (range 37 to 67 years). Demographic and clinical characteristics in two groups, before and after treatment were shown in Table 1. 

**Table 1:** Demographic information of patients in the two groups

Parameter	Groups*	
	Nystatin	ZM
Age (yrs.)		
mean± SD	60.93±13.04	55.86±10.04
Median (range)	38-82	37-67
Gender		
Female	5 (35.7%)	2 (14.3%)
Male	9 (64.3%)	12 (85.7%)
Denture stomatitis type		
II	7 (50 %)	7 (50 %)
III	7 (50 %)	7 (50 %)
Duration of wearing a denture		
<5(yrs)	2(14.3%)	2(14.3%)
>5(yrs)	12(85.7%)	12(85.7%)
Cleaning method		
Water	4(28.6%)	2(14.3%)
Tooth brush	10(71.4%)	12(85.7%)
Cleaning agent	0(0%)	0(0%)
Frequency of cleaning		
None	0(0%)	0(0%)
1 qw	3(21.4%)	0(0%)
2 qw	1(7.1%)	3(21.4%)
3 qw	0(0%)	3(21.4%)
>3 qw	10(71.4%)	8(57.1%)
Nocturnal denture use		
No	2(14.3%)	1(7.1%)
Yes	12(85.7%)	13(92.9%)

* Statistical analysis was revealed no significant difference (*p*> 0.05)

**Figure 2:**
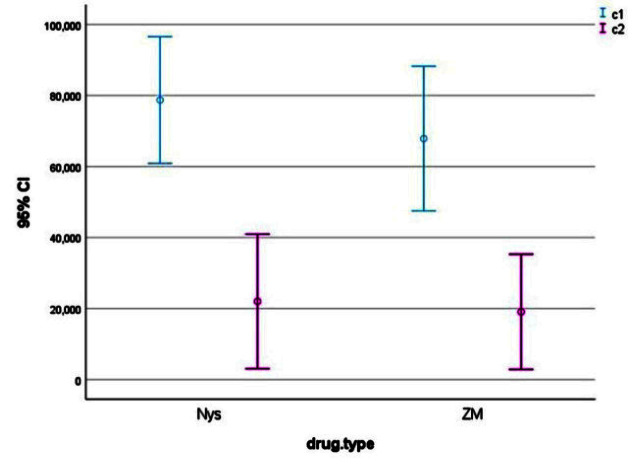
Colony count (CFUs) before and after treatment in the two groups

**Figure 3:**
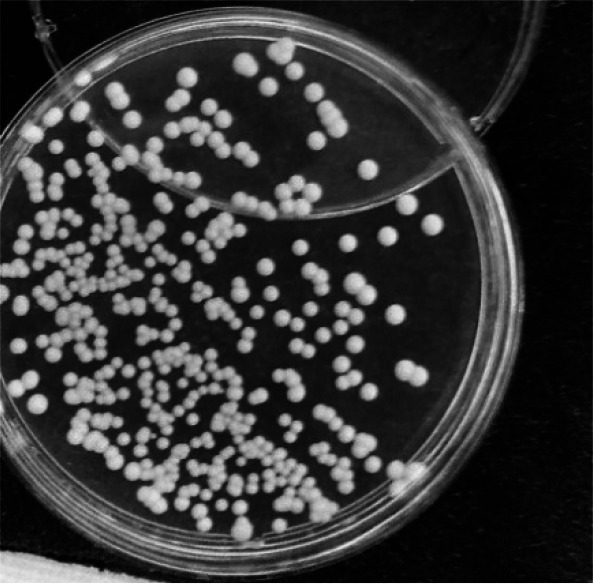
*Candida albicans* colony forming units before treatment with ZM

**Figure 4:**
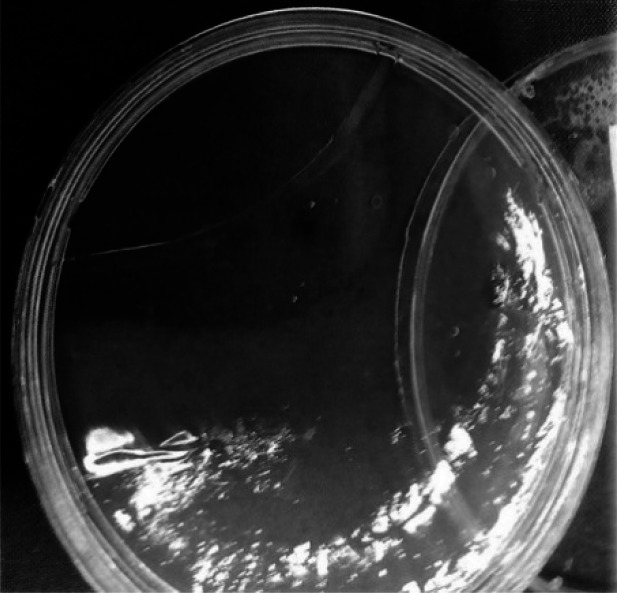
*Candida albicans* colony forming units after treatment with ZM

**Figure 5:**
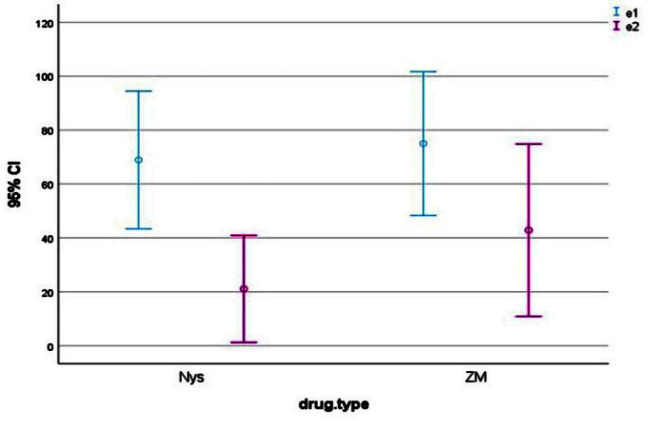
Erythema before and after treatment in the two groups

**Figure 6:**
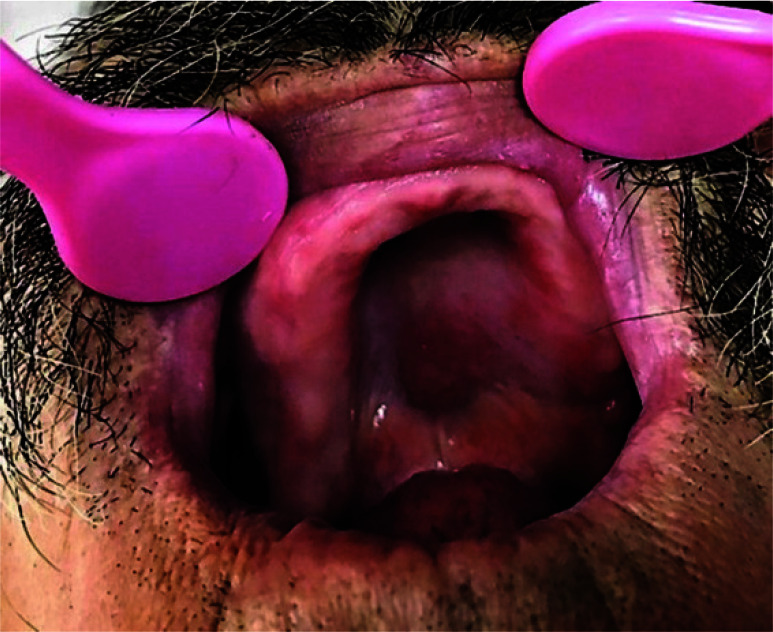
Erythema before treatment with nystatin

**Figure 7:**
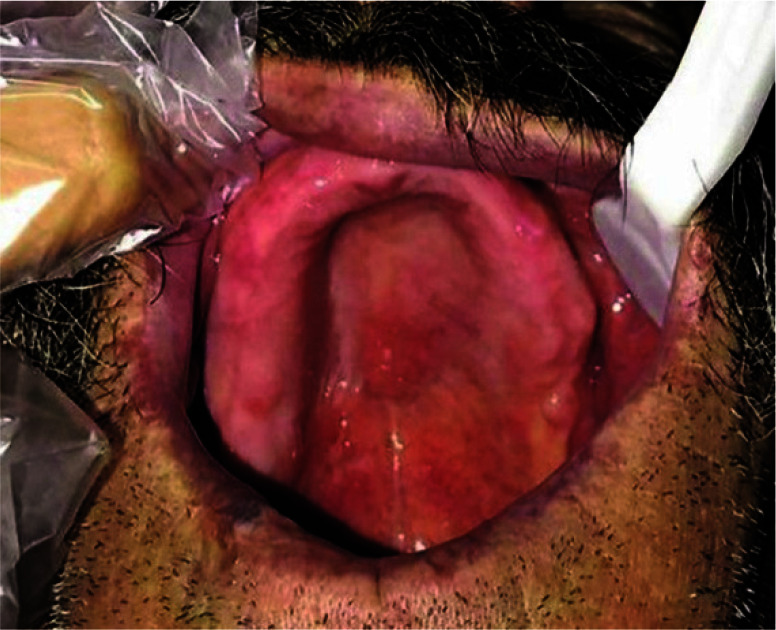
Erythema after treatment with nystatin

**Figure 8:**
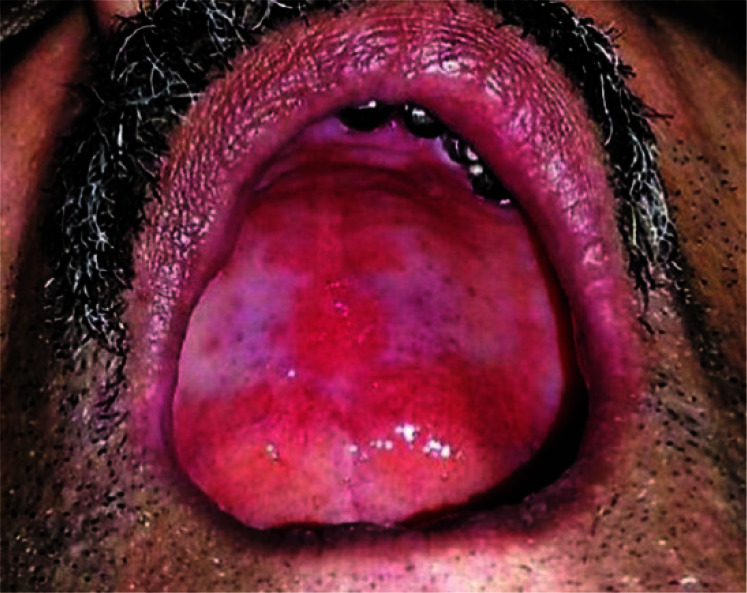
Erythema before treatment with ZM

**Figure 9:**
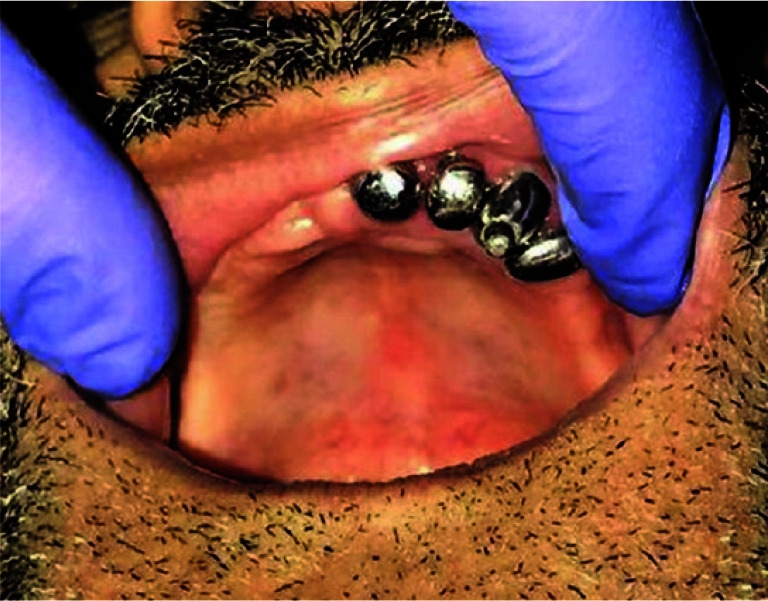
Erythema after treatment with ZM

Patients in the case and control groups were compared in terms of *C. albicans* colony count at baseline and 14 days after treatment. The results showed that ZM and nystatin had no significant difference with each other in this regard (*p*= 0.593) and both caused a significant reduction of *C. albicans* colony count (*p*< 0.001, Table 2, Figures 2-4). Comparison of erythema before and at 14 days after treatment revealed that both nystatin and ZM significantly decreased the erythema (*p*< 0.001) and both were equally effective in this regard with no significant difference with each other (*p*= 0.256; Table 3, Figures 5-9).

**Table 2:** Colony count (CFUs) before and after treatment in the two groups

	Groups		*p* Value
	Nystatin	ZM	
CFU (before)			*p*= 0.593
Min	15000	20000	
Max	100000	100000	
mean±SD	78714.29±	30897.91	
	67857.14±	35285.33	
CFU (after)			
Min	0	0	
Max	80000	100000	
mean±SD	22000±	32825.88	
	19071.43±	28089.04	
*p* value	*p*< 0.001		

SD: Standard deviation

**Table 3:** Erythema before and after treatment in the two groups

	Groups		*p* Value
	Nystatin	ZM	
Erythema (before)(mm2)			*p*= 0.256
Min.	10	20	
Max.	150	170	
Mean± SD	68.93±44.25	75±46.24	
Erythema (after) (mm2)			
Min	0	0	
Max	100	170	
Mean± SD	21.07±34.42	42.86±55.39	
*p* Value	*p*< 0.001		

SD: Standard deviation

## Discussion

The current findings revealed similar efficacy of nystatin and ZM in the reduction of *C. albicans* colony count (*p*= 0.593) and they both caused a significant reduction in *C. albicans* CFUs (*p*< 0.001).

The same results were obtained for resolution of erythema and both medications equally decreased erythema (*p*< 0.001) with no significant difference with each other (*p*= 0.256).

Amanlou *et al*. [ 12 ] compared the efficacy of miconazole and ZM gel for the treatment of denture stomatitis in a clinical trial. Similarly, in our study, erythema significantly decreased in both groups with no significant difference between them. A reduction was also noted in *C. albicans* colony count on day 14 in both groups with no significant difference between nystatin and ZM in this respect. However, Amanlou *et al*. [ 12 ] showed superior efficacy of miconazole in this respect. This difference in the results can be due to the differences in the efficacy of nystatin and miconazole and different forms of medications used (gel versus drop). 

Sajed *et al*. [ 8 ] performed a systematic review of ethnopharmacology, pharmacology, toxicity, modern pharmaceutical applications, and pharmacochemistry of ZM. They reviewed all relevant articles published until 2012 and concluded that ZM has antimicrobial, antioxidative, anti-inflammatory, antispasmodic, and analgesic properties. ZM oil contains high concentrations of oxygenated monoterpenes, thymol, and carvacrol and possesses favorable antimicrobial properties. Our study also confirmed the anti-candida effects of ZM. 

Moghim *et al*. [ 5 ] evaluated the antifungal effects of ZM and Nigella sativa on *C. albicans*. They measured the minimum inhibitory concentration (MIC), 50% MIC, 90% MIC, and minimum fungicidal concentration (MFC) of ZM and Nigella sativa separately by counting the fungal colonies. The results revealed that both ZM and Nigella sativa were effective against C.albicans (*p*< 0.005). This finding was in agreement with our results. 

Jafari *et al*. [ 17 ] evaluated the antifungal effects of ZM essence on acrylic resin plates contaminated with *C. albicans*. In their in vitro experimental study, they compared five different concentrations of ZM essence (3.125 mg/mL to 50mg/mL), 100,000-unit nystatin as the positive control, and saline as the negative control. They concluded that ZM essence in 25mg/mL and 50mg/mL concentrations had a MFC similar to that of nystatin and eliminated 100% of the *C. albicans* colonies. Similarly, ZM in our study caused a significant reduction in *C. albicans* colony count. 

Sedigh-Shams *et al*. [ 18 ] compared the antifungal effects of sodium hypochlorite and ZM essence as irrigating solutions for root canals contaminated with *C. albicans* in vitro. They first calculated the MFC of ZM and sodium hypochlorite. The results showed that sodium hypochlorite in its MFC and ZM in twice its MFC had the highest antifungal effect with no significant difference with each other (*p*> 0.05). However, their antifungal effects were significantly different from those of ZM in MFC and distilled water (*p*< 0.05). Their findings confirmed the antifungal effect of ZM and were in line with our results.

Khosravi *et al*. [ 19 ] compared the effects of ZM essential oil and itraconazole on disseminated *C. albicans* infection in rats. They administered 30mg/mL, 48mg/ mL, and 64mg/mL of the essential oil of ZM and 200mg/mL itraconazole intraperitoneally. The results showed that injection of 64 mg/mL essential oil of ZM had the highest efficacy for the reduction of *C. albicans* colony count and itraconazole was less effective for this purpose (*p*< 0.01 for the brain, *p* < 0.0005 for the lungs, and *p*< 0.0005 for the kidneys). However, itraconazole was more effective than 30 mg/mL concentration of ZM for the elimination of *C. albicans* in the brain (*p*< 0.02), kidneys (*p*< 0.02), and spleen (*p*< 0.04). No significant difference was noted between itraconazole and 48 mg/mL concentration of ZM. The difference between their results and ours regarding the efficacy of ZM can be due to some reasons. First, dissimilarities between human and animal models, second, diversities in types of *C. albicans* infection, and finally differences between ZM concentrations. 

Fouladi *et al*. [ 20 ] compared the efficacy of ZM cream with vaginal clotrimazole cream for the treatment of vaginal candidiasis in 73 patients. They reported that 1% clotrimazole cream and ZM both caused a significant improvement in patients (*p*< 0.05). Their findings were in agreement with our results. 

Considering all the above and the current results, it may be concluded that ZM is effective for the reduction of *C. albicans* colony count due to its antifungal and anti-inflammatory properties. 

## Conclusion

As a potent antifungal drug, ZM may be as effective as nystatin for the reduction of erythema of the palate and 

*C. albicans* colony count.
